# Foreign cultural norms are better accepted in the second language

**DOI:** 10.1111/nyas.15407

**Published:** 2025-08-04

**Authors:** Jiehui Hu, Aina Casaponsa, Wanyu Zhang, Rafał Jończyk, Yan Jing Wu, Shan Gao, Guillaume Thierry

**Affiliations:** ^1^ School of Foreign Languages University of Electronic Science and Technology of China Chengdu China; ^2^ Ministry of Education Key Lab for Neuroinformation, University of Electronic Science and Technology of China Chengdu China; ^3^ Department of Linguistics and English Language Lancaster University Lancaster UK; ^4^ School of Foreign Languages and Cultures Nanjing Normal University Nanjing China; ^5^ Faculty of English Adam Mickiewicz University Poznan Poland; ^6^ Cognitive Neuroscience Center Adam Mickiewicz University Poznan Poland; ^7^ School of Linguistics Sciences and Arts Jiangsu Normal University Jiangsu China; ^8^ School of Psychology and Sport Science Bangor University Bangor Wales UK

**Keywords:** bilingualism, cultural norms, event‐related potentials, N400, semantic processing

## Abstract

Cultural diversity goes hand‐in‐hand with language variation. It is unknown however, whether using a second language influences one's disposition toward cultural concepts. Here, we show that bilinguals process violations of cultural norms differently depending on whether concepts are introduced in the native (L1) or the second (L2) language. Participants read sentences that were acceptable or not, independent of culture, or acceptable in one culture but not in the other. Culture‐independent generic knowledge was integrated similarly across languages, as indexed by N400 modulations of event‐related brain potentials, whereas statements conforming to British culture were better accepted by Chinese–English bilinguals when presented in L2 English than L1 Chinese. To our knowledge, these findings offer the first evidence for an effect of language of operation on cultural judgments in bilinguals. Functioning in a second language thus disposes one to be more tolerant toward foreign cultural values, which has important implications in a culturally diverse world.

## Graphical abstract

Using behavioral and brain potential measures, we tested Chinese–English bilinguals’ acceptance of statements. While participants processed culture‐nonspecific generic knowledge similarly in their L1 Chinese and L2 English, the violation effect elicited by British culture statements was abolished when they were read in L2 English. These findings offer the first evidence that functioning in a second language disposes one to better accept foreign cultural norms.

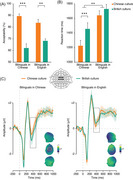

## INTRODUCTION

Language is intrinsically linked with culture in multiple and complex ways.^1^, ^2^, ^3^, ^4^ People efficiently express ideas or describe events referring to generic knowledge that is shared—or not—with others.^5^, ^6^ Moreover, the way language is used and understood is strongly constrained by the cultural context in which we live.^7^, ^8^, ^9^ This observation has led to the proposal that language represents “a guide to understanding a community's world view”^10^ beyond a simple, basic understanding of situations.

Despite variations in cultural norms, members of different communities are able to communicate efficiently when they are proficient in the language of their interlocutor. Everyone knows that breathing is essential to life, for instance. Thus, a sentence starting with “She stopped breathing entirely and*…*” implies a negative ending in any language. The same, however, is not always true of culture‐specific information. When Princess Kate, the Duchess of Cambridge, presented her new born baby to the public, purportedly 10 h postpartum, the official photographs sent a shockwave through the Chinese community because many were in awe that a mother could be seen outside, standing in heels, wearing makeup, and displaying her newborn in the open so soon after giving birth.^11^ A new mother in China is expected to stay in bed for several days without washing her body (a form of postnatal confinement called *zuo yue zi*) and she will traditionally be served chicken soup or a hot drink to help her recover from labor. A British mother, in stark contrast, may choose to take a shower merely a few hours after giving birth, is likely to drink cold water, and is expected to leave maternity care within 24 h, all of which is inconceivable in Chinese culture. Inasmuch as parties involved in an act of communication can often be led to adopt the same mind‐set, culture‐specific situations can yield radically different responses in speakers of different languages.^12^, ^13^

Bi‐ and multicultural individuals who have been acquainted with two or more cultures can shift between different cultural frames when presented with effective contextual cues.^14^, ^15^ This evidence has been taken to argue that bilinguals can separately internalize two different pools of domain‐specific knowledge and selectively activate or suppress them depending on the situation.^14^ Among the cues enabling such shifts, language appears to have a critical tuning effect. For instance, people have been shown to abandon old cultural preferences in favor of new ones aligned with a newly learned language.^3^, ^16^, ^17^ However, these studies cannot determine whether language‐induced cultural shifts represent overt, strategic choices between culture‐specific codes or derive from changes in semantic representations. It thus remains unknown whether cultural shifts are motivated by deliberate, pragmatic social conformism, or are constrained by language‐bound conceptual changes. A mechanistic account of the phenomenon thus requires measurements that tap into unconscious and automatic aspects of information processing in the human brain (e.g., see Ellis et al.^18^).

Here, using the N400 component, a robust index of semantic processing derived from brain activity, we tested the hypothesis that cultural expectations can change as the result of language of operation at a semantic level, after establishing that generic semantic integration is unaffected by language of operation. This approach focused our results on implicit processing, given that the task required semantic acceptability judgments based on world knowledge rather than evaluation of cultural norms. We recorded scalp electrical activity in fluent Chinese–English bilinguals while they read statements such as “Upon giving birth, she was advised to take a bath after a…” ending in one of four possible ways (see Table 1): (a) acceptable, that is, nonculture specific and compatible with generic knowledge (e.g., “while”); (b) unexpected, that is, possible but highly unlikely in any cultural context (“year”); (c) more compatible with Chinese culture (“week”); or (d) more compatible with British culture (“few hours”). Critically, we ensured that sentence endings representative of a given culture were unacceptable from the perspective of the other culture. We used the N400, a negative modulation of event‐related brain potentials (ERPs) peaking at around 400 ms poststimulus^19^, ^20^ as an objective index of participants’ conceptual processing of sentence endings in the four conditions.

**TABLE 1:** Experimental conditions and examples of stimuli.

Test	Example sentence	Ending	Acceptability	Language
**A**. Generic knowledge	Upon giving birth, she was advised to take a bath after a …	while.	Acceptable	English
		year.	Unexpected	
	刚生了小孩后, 她被建议洗澡需要等	一会儿。	Acceptable	Chinese
		一年。	Unexpected	
**B**. Cultural acceptability	Upon giving birth, she was advised to take a bath after a …	week.	Chinese culture	English
		few hours.	British culture	
	刚生了小孩后, 她被建议洗澡需要等	一周。	Chinese culture	Chinese
		几小时。	British culture	

Overall, we expected semantic processing to be more cognitively costly—and thus the N400 to increase in amplitude—when a sentence completion was unexpected as compared to when it was acceptable, irrespective of participants’ cultural background. *Prediction 1*: Both fluent Chinese–English bilinguals and a control group of English native speakers should demonstrate comparable processing of statements probing culture‐independent generic knowledge. *Prediction 2*: Processing of generic statements should not be modulated by language of operation in bilinguals given their good command of English. *Prediction 3*: We should observe diametrically opposed effects in Chinese and British participants operating in their native language when statements convey situations in radical contrast between Chinese and British culture. N400 amplitude modulations should thus be reversed between groups for this contrast. *Prediction 4*: Assuming that language of operation affects the way in which culture‐specific knowledge is processed at a semantic level, we expect a change of language to alter the pattern of N400 modulation elicited by cultural norm violations in bilinguals. While Predictions 1, 2, and 3 served as reality checks, validating both the generic knowledge and culture‐specific stimuli used in this study, Prediction 4 is not trivial, given the notorious resilience of the N400 to task manipulations and its automatic nature.^19^, ^21^ We found a critical interaction between language of operation and cultural norm on both the N400 amplitudes and acceptability judgments in bilinguals, suggesting a fundamental difference between culture‐specific and generic semantic knowledge in the human mind, and showing that cultural shifts in bicultural individuals stem from implicit semantic representations rather than explicit, strategic behavioral control.

## METHODS

### Participants

We tested 20 late, fluent Chinese–English bilinguals (six males, 14 females; mean = 25.1 years, SD = 4.2) and 20 native speakers of English (nine males, 11 females; mean = 20.1 years, SD = 3.6) at Bangor University, Wales. All Chinese–English bilinguals had been living in the United Kingdom for an average duration of 1 year and 10 months (SD = 3.6 months) and reported using on average 60% Chinese and 40% English in everyday interactions. Their self‐reported English proficiency on the International English Language Testing System (https://ielts.org) band score (from 1 to 9) was 7.1 on average (range 6–8.5). British participants declared having been born in the United Kingdom. English was their native language and they had no experience of learning Chinese. Ethical approval was granted by the School of Psychology, Bangor University ethics committee, and participants gave written consent before taking part. They were either given five course credits or compensation in cash for their time.

### Stimuli and procedure

A total of 46 English sentence sets and corresponding Chinese equivalents were constructed for each of four experimental conditions: Acceptable or unexpected from a generic knowledge viewpoint; acceptable according to Chinese culture but unexpected in British culture; or vice versa. Sentence onsets were repeated between conditions, and thus only differed in their endings. The four endings for each sentence context featured one to three words in order to unambiguously convey the meaning of the statement, while keeping the sentence beginning constant (e.g., “few hours” in the cultural acceptability statement in Table 1). Equivalent statements in English and Chinese were created on the basis of back‐and‐forth iterative translations until the expressions in Chinese and in English were stable, and sounded fluid, natural, and grammatical to native speakers of either languages (see Liversedge et al.^22^ for a similar approach), while keeping the critical word cluster in sentence–final position. The 46 initial sentence sets were then submitted to four rounds of stimulus norming in more than 20 native speakers of Chinese in China (20–25) and native speakers of English (20–23) in the United Kingdom in each round. Different participants in each of the norming cycles were instructed to rate the semantic acceptability of sentence completions on a Likert scale from −2 (totally unacceptable) to 2 (totally acceptable, i.e., a simplified index of Cloze probability). Adjustments were made to the stimuli after each norming cycle and sentences eliciting unanticipated or unreliable ratings were discarded, leading to a final experimental set of 38 sentences. Sentence–final word clusters did not significantly differ between conditions in terms of keyword written frequency, number of words, and length in syllables [English: CELEX lexical database;^23^ Chinese: Modern Chinese Corpus Wordlist,^24^ all *p*s > 0.1]. Acceptability ratings for the final set are provided in the lower row of Table S1 (round 4).

**FIGURE 1:**
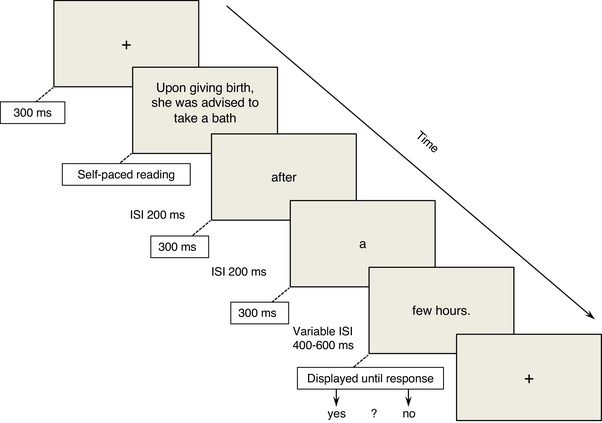
Structure of an experimental trial and timing parameters. ISI, interstimulus interval.

Stimuli were presented in 18‐point white courier new font on a black background in the center of a 19″ monitor with a refresh rate of 75 Hz. The bulk of each sentence was presented all‐at‐once and reading was self‐paced (Figure 1). Upon pressing the button, one‐to‐three penultimate words were presented one word at a time in a rapid serial visual presentation stream in the center of the screen, so as to minimize contamination of electrophysiological recordings by eye movements, each for a duration of 400 ms with an interstimulus interval (ISI) of 200 ms. The sentence–final word cluster was then presented all at once and clearly identifiable as final because it ended with a full stop. It was displayed after a variable ISI (randomly selected between 400 and 600 ms in steps of 20 ms) to minimize ERP contamination from the penultimate stimulus word. Final word clusters contained 7.2 (±2.5) letters on average and presented with two degrees of visual angle to prevent eye saccades. Following the presentation of a sentence ending, participants indicated whether or not the sentence was semantically acceptable (i.e., whether the sentence made sense according to world knowledge) by pressing designated buttons on a response box. Sentences were presented in four blocks per language in a pseudorandomized order. No sentence onset was repeated within a given block. Block order and response keys were rotated between participants. Chinese–English bilinguals were tested in eight blocks, four in English, four in Chinese, and language order was counterbalanced. Another 32 filler sentences were built according to the same rules as the experimental stimuli and distributed equally among the four testing blocks (four acceptable sentences and four unacceptable sentences per experimental block). Filler sentences had random cultural acceptability and cloze probability.

### ERP recording and processing

Electrophysiological (EEG) data were recorded from 64 Ag/AgCl electrodes positioned according to the extended 10–20 convention, in reference to Cz at a rate of 1 kHz. Accuracy and reaction times were recorded simultaneously to EEG activity. All impedances were kept below 5 kΩ. EEG recordings were filtered online using a band‐pass filter between 0.05 and 200 Hz. Preprocessing steps and analyses were performed using MATLAB (R2022a, The Mathworks, Inc.) and a combination of scripts and routines implemented in the EEGLAB (v.2024.0)^25^ and ERPLAB (v.10.02)^26^ toolboxes. The data were refiltered off‐line with a 30‐Hz lowpass using Finite Impulse Response filter, and referenced to the global average reference. Ocular artifacts were corrected via independent component analysis (ICA) using pop_runica function. The resulting ICA decomposition was inspected using IClabel.^27^ Independent components containing ocular artifacts, bad channels activity and/or line noise were removed from the data (*M*_IC removed‐bilinguals_ = 2.8, SD = 1.3; *M*_IC removed‐English natives_ = 2.4, SD = 0.88). Artifacts exceeding ±100 µV were automatically discarded. There was a minimum of 30 valid trials per condition in every participant (see Table S2 for more details). Epochs ranged from −200 to 1000 ms after the onset of the sentence completion. Baseline correction was applied in reference to prestimulus activity. N400 mean amplitudes were measured in the time‐window of predicted differences (300–500 ms) at electrodes of expected maximal modulation (FC1, FC2, FCz, C1, C2, Cz) for cultural information. P600 mean amplitudes were measured in the time‐window of predicted differences (550–700 ms) at electrodes of expected maximal modulation (CP1, CPz, CP2, P1, Pz, P2).

### Data analysis

Behavioral analyses were conducted using the *lme4* package^28^ in R. Continuous outcome measures (reaction times) were analyzed with linear mixed‐effect models (*lme*). Significant *p* values and type III *F*‐statistics for main effects and interactions were calculated using Satterthwaite approximations to denominator degrees of freedom as implemented in the *lmerTest* package.^29^ Planned comparisons and *β* estimates of main effects and interactions were calculated via contrasts of least squared means as implemented in *difflsmeans* and *lsmeans* of the *lmerTest* package. Binary outcomes (acceptability judgments with 1 indicating true statement and 0 false statement) were analyzed using mixed‐effects logit models.^30^ Type III Wald *χ* ^2^‐statistics for main effects and interactions were calculated using the *car* package.^31^ Planned comparisons and *β* estimates of main effects and interactions were calculated via contrasts of least squared means as implemented in *lsmeans* of the *car* package. Note that type III Wald *χ* ^2^‐statistics for main effects in mixed‐effects logit models are sensitive to the reference level used in the interaction term, hence least squared means differences are also reported when appropriate. All models included maximal within‐unit random effects structure,^32^, ^33^ thus random intercepts and relevant random slopes for all the within‐unit variables and interactions were included for each prediction tested. Since all participants conducted four blocks of pseudorandomly selected sentences per language during the individual sessions, this factor (i.e., block) was included as a control variable in the fixed‐effect structure of all models.^33^ Response latencies that deviated more than three interquartile range above the third and below the first quartile of each intraparticipant and intraitem factors were considered outliers and discarded from all behavioral analyses (within‐participant = 2.20%; within‐item = 2.39%; total = 4.21%).

ERP data were submitted to analyses of variance (ANOVA) for each of the four predictions using the mean amplitudes of the N400 as a dependent variable. All analyses followed a two‐by‐two repeated measures design. Prediction 1 and 3 included within‐ and between‐participant factors, while Predictions 2 and 4 included only within‐participant factors (see Supporting Information Results for further specifications of each individual analysis). Type III *F*‐statistics for main effects and interactions are reported as well as *p* values and effect sizes calculated using partial eta squared. Planned comparisons were calculated using two‐tailed *t*‐tests and assuming equal variance across samples. Conventional statistical hypothesis testing can only provide evidence against the null hypothesis due to unknown probability of type II error, with a probability of 5% of being incorrectly rejected (type I error). To address this issue, we conducted Bayes factor analyses in addition to the main analyses for each individual prediction.^34^ Bayes factor is an alternative to conventional *t*‐tests that allows us to test whether the data favor more the null hypothesis or the alternative. We report BF_01_ for results favoring the null hypothesis and BF_10_ for results favoring the alternative hypothesis. To conduct these analyses, we used the *BayesFactor* package in R.^35^

## RESULTS

**FIGURE 2:**
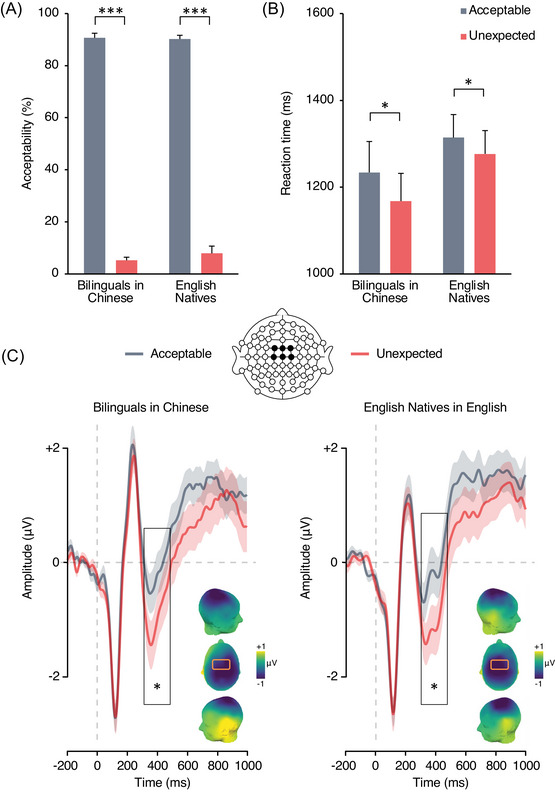
Comparison of behavioral performance across groups in the test of generic semantic knowledge. (A) Acceptability rates. (B) Reaction times. Error bars depict SEM. (C) Event‐related potentials elicited by sentence–final word clusters over the frontocentral area (linear derivation of FC1, FCz, FC2, C1, Cz, C2) in bilinguals processing generic knowledge in Chinese (left) and English native controls processing generic knowledge in English (right). Significant differences are highlighted as follows: *p < 0.05, **p < 0.01, and ***p < 0.001.

First, we tested the assumption that generic semantic violations would yield comparable effects in native speakers of Chinese and native speakers of English (Prediction 1). Chinese–English bilingual participants read sentences in Chinese that ended in a semantically acceptable or unexpected fashion and British participants read the same statements in English (Table 1A). As expected, acceptability rates were high for acceptable endings (89%; *β* = 3.14, SE = 0.36) and low for endings violating generic semantic knowledge (6%; *β* = –4.49, SE = 0.45). This resulted in a significant main effect of acceptability, in the absence of a group effect or an interaction between acceptability and group (see Supporting Information Results). Thus, generic knowledge statements presented in participants’ L1 (either Chinese or English) were rated as acceptable or unexpected by Chinese and British participants alike (Figure 2A). Participants were slower to characterize a sentence ending as acceptable (*β* = 1356, SE = 70) than unexpected (*β* = 1194, SE = 59), in the absence of an effect of group or an interaction (Figure 2B).

As predicted, N400 mean amplitude was significantly more negative for sentence endings violating generic semantic knowledge as compared to acceptable endings (*F*_1, 38_ = 20.25, *p* < 0.001, $\eta_{p}^{2}$ = 0.35; Figure 2C). There was no effect of group (*F*_1, 38_ = 0.002, *p* = 0.96, $\eta_{p}^{2}$ < 0.001) and no interaction (*F*_1, 38_ = 0.04, *p* = 0.84, $\eta_{p}^{2}$ < 0.001). Bayes factor analyses of the magnitude of the N400 modulation by generic knowledge violation between groups showed that the data were three times more probable under the null hypothesis than the alternative (BF_01_ = 3.17).

Prediction 1 was thus validated by behavioral and electrophysiological data: Both groups showed similarly lower acceptability rates, faster reaction times, and greater N400 amplitude for unexpected than acceptable generic knowledge statements.

### Comparable generic knowledge processing between languages in bilinguals

We then employed a within‐subject analysis to test whether a change of language would affect semantic processing of generic knowledge in Chinese–English bilinguals. Participants read equivalent statements with semantically acceptable or unexpected endings separately in Chinese and English blocks (Table 1A).

**FIGURE 3:**
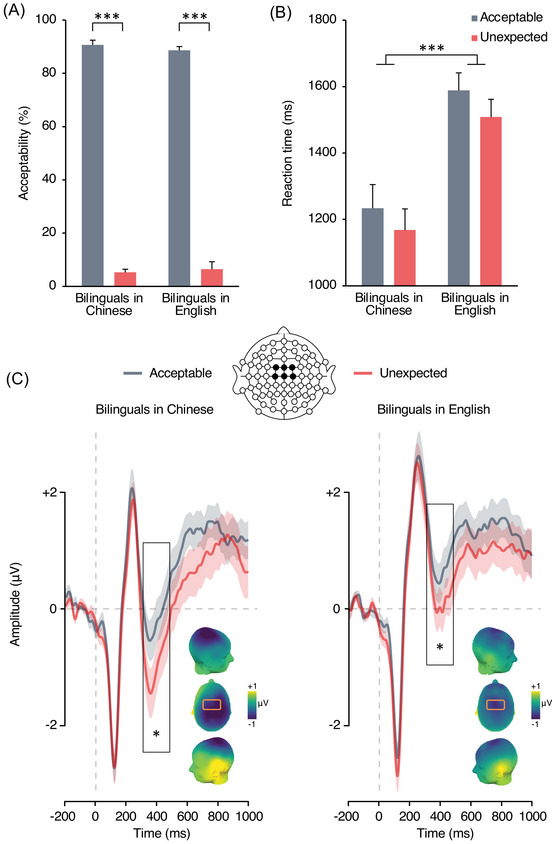
Comparison of behavioral performance across languages within Chinese–English bilinguals in the test of generic knowledge. (A) Acceptability rates. (B) Reaction times. Error bars depict SEM. (C) Event‐related potentials elicited by sentence–final word clusters over the frontocentral area (linear derivation of FC1, FCz, FC2, C1, Cz, C2) in bilinguals processing generic knowledge in Chinese (left) and in English (right). Significant differences are highlighted as follows: *p < 0.05, **p < 0.01, and ***p < 0.001.

As anticipated, acceptability rates were high for acceptable endings (88%; *β* = 3.11, SE = 0.41) and low for generic knowledge violations (6%; *β* = –4.54, SE = 0.51; Figure 3A). This resulted in a significant main effect of acceptability, no effect of language, and no interaction between acceptability and language of operation (see Supporting Information Results). Participants responded faster in Chinese (*β* = 1239, SE = 84) than English (*β* = 1697, SE = 95). They were slower to characterize a sentence ending as acceptable (*β* = 1567, SE = 95) than unexpected (*β* = 1372, SE = 73), and there was no interaction (Figure 3B).

N400 mean amplitude analysis revealed a significant main effect of language of operation (*F*_1, 19_ = 18.51, *p* < 0.001, $\eta_{p}^{2}$ = 0.49) and of acceptability (*F*_1, 19_ = 13.90, *p* = 0.001, $\eta_{p}^{2}$ = 0.42), but no interaction between the two factors (*F*_1, 19_ = 0.65, *p* = 0.43, $\eta_{p}^{2}$ = 0.03; Figure 3C). Hence, while reading in Chinese elicited more negative N400 amplitudes than reading in English, N400 mean amplitude was consistently more negative for generic knowledge violations than semantically acceptable sentences, irrespective of language. Bayes factor analyses of acceptability differences across languages favored the null hypothesis, such that the absence of a difference between languages was around three times more probable than the alternative (BF_01_ = 2.97).

Thus, results validated Prediction 2: While Chinese–English bilinguals displayed faster reaction times and greater N400 amplitude when reading in Chinese, differences between acceptable and unexpected endings were unaffected by language of operation.

### Opposite cultural expectations between groups

Next, we turned to culture‐dependent information processing. Chinese and British participants read sentences representative of either Chinese or British culture in their native language (Table 1B). We tested the assumption that acceptability judgments and associated N400 modulations would be reversed between groups given the contrast between Chinese and British cultural norms exploited in the experiment.

**FIGURE 4:**
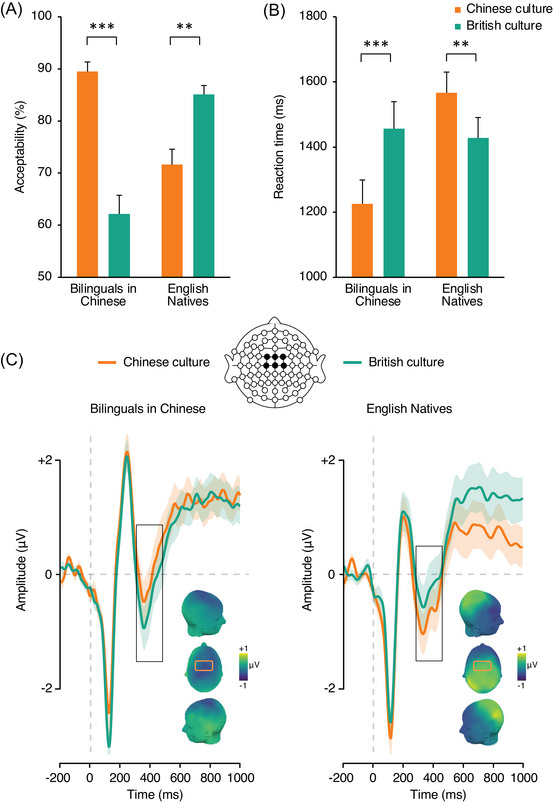
Comparison of behavioral performance across groups in the test of cultural norms. (A) Acceptability rates. (B) Reaction times. Error bars depict SEM. (C) Event‐related potentials elicited by sentence–final word clusters over the frontocentral area (linear derivation of FC1, FCz, FC2, C1, Cz, C2) in bilinguals processing cultural norms in Chinese (left) and English native participants processing cultural norms in English (right). Significant differences are highlighted as follows: *p < 0.05, **p < 0.01, and ***p < 0.001.

As predicted, we found a significant interaction between cultural norm and group, such that English speakers rated statements representative of the British culture as more acceptable than statements representative of the Chinese culture (*β* = 1.14, SE = 0.44, *z* = 2.60, *p* = 0.009; British culture: 83%, *β* = 2.37, SE = 0.35; Chinese culture: 69%, *β* = 1.23, SE = 0.36), whereas Chinese speakers rated statements representative of the Chinese culture as more acceptable than statements representative of the British culture (*β* = 2.40, SE = 0.41, *z* = 5.85, *p* < 0.001; Chinese culture: 89%, *β* = 3.08, SE = 0.38; British culture: 61%, *β* = 0.69, SE = 0.30; Figure 4A). There was also a main effect of culture on acceptability rates, which were higher for statements representative of the Chinese culture (79%, *β* = 2.16, SE = 0.30) than for statements representative of the British culture (73%, *β* = 1.53, SE = 0.26). We also found a main effect of group (see Supporting Information Results). Response time analyses mirrored the crossed interaction between group and cultural norm but the main effects of group and cultural norm were not significant (Figure 4B).

N400 mean amplitude analysis revealed no main effect of group (*F*_1, 38_ = 0.12, *p* = 0.72, $\eta_{p}^{2}$ = 0.001) or cultural norm (*F*_1, 38_ = 0.03, *p* = 0.87, $\eta_{p}^{2}$ = 0.001), but a significant interaction of the two factors (*F*_1, 38_ = 8.26, *p* = 0.007, $\eta_{p}^{2}$ = 0.18). Planned one‐tailed comparisons showed that sentence endings representative of British culture elicited greater N400 mean amplitude than Chinese culture endings when bilingual participants read in Chinese (*t*_38_ = −2.15, *p* = 0.019). In contrast, sentence endings representative of Chinese culture elicited significantly increased N400 mean amplitudes than British culture endings in British participants (*t*_38_ = −1.91, *p* = 0.032; Figure 4C). Bayes factor analyses comparing the magnitude of cultural norm violation effect between groups favored the alternative hypothesis (i.e., a markedly different response to cultural norm violation between ethnic groups), BF_10_ = 7.48.

### Embracing foreign culture in the second language

Finally, we tested the critical assumption that culture‐specific knowledge may be processed differently depending on language of operation. We expected sentences representative of Chinese and British culture, respectively, to elicit different patterns of acceptability rates, reaction times, and N400 amplitude modulations when Chinese–English bilinguals operate in their second language.

**FIGURE 5:**
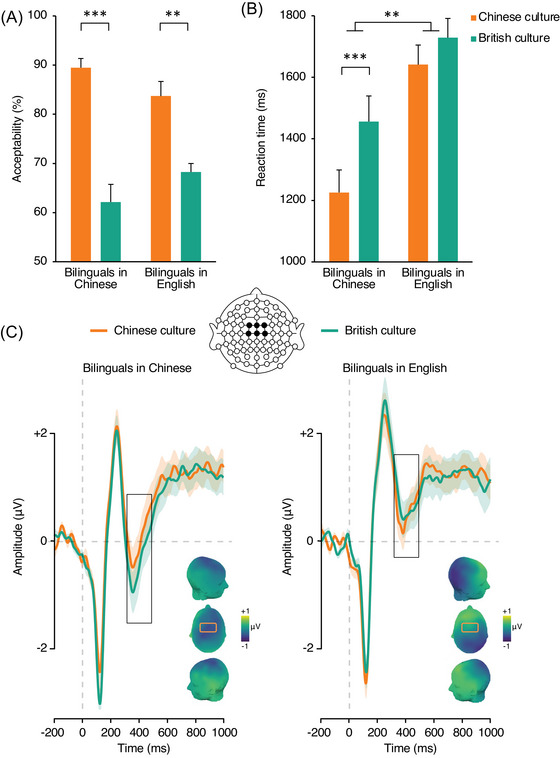
Comparison of behavioral performance across languages in Chinese–English bilinguals in the test of cultural norms. (A) Acceptability rates. (B) Reaction times. Error bars depict SEM. (C) Event‐related potentials elicited by sentence–final word clusters over the frontocentral area (linear derivation of FC1, FCz, FC2, C1, Cz, C2) in bilinguals processing cultural norms in Chinese (left) and in English (right). Significant differences are highlighted as follows: *p < 0.05, **p < 0.01, and ***p < 0.001.

We found a significant interaction between cultural norm and language on acceptability ratings. Post hoc analyses revealed that English‐culture statements were rated as more acceptable when presented in English than when presented in Chinese (*β* = 0.39, SE = 0.17, *z* = 2.33, *p* = 0.02; English context: 67%, *β* = 1.08, SE = 0.33; Chinese context: 61%, *β* = 0.69, SE = 0.31), while Chinese‐culture statements were rated as more acceptable when presented in Chinese than in English (*β* = 1.02, SE = 0.47, *z* = 2.16, *p* = 0.03; Chinese context: 89%, *β* = 3.33, SE = 0.58; English context: 83%, *β* = 2.32, SE = 0.37). Bayes factor analyses confirmed that the data were around 10 times more probable under the alternative hypothesis (i.e., difference between languages of operation) for both British culture‐specific statements (BF_10_ = 10.78) and Chinese‐culture‐specific statements (BF_10_ = 10.75). Acceptability rates were overall significantly higher for statements following Chinese cultural norms (86%, *β* = 2.83, SE = 0.42) as compared to British cultural norms (64%, *β* = 0.89, SE = 0.3; *χ* ^2^_1_ = 8.42, *p* = 0.004; Figure 5A) and we found no other effect (see Supporting Information Results).

Reaction time analyses revealed a main effect of cultural norm (*F*_1, 76.29_ = 12.83, *p* < 0.001) and language of operation (*F*_1, 20.19_ = 9.88, *p* = 0.005) in the absence of a significant interaction (*F*_1, 31.39_ = 2.64, *p* = 0.11). As expected, participants’ responses were faster in Chinese (*β* = 1456, SE = 118) than in English (*β* = 1950, SE = 128) and for Chinese cultural norms (*β* = 1560, SE = 97) than English cultural norms (*β* = 1845, SE = 108; Figure 5B).

Critically, when comparing N400 amplitude modulations by cultural norm and language of operation in the same group of participants, we found a main effect of language (*F*_1, 19_ = 6.96, *p* = 0.017, $\eta_{p}^{2}$ = 0.27) and a significant interaction between language and cultural norm (*F*_1, 19_ = 5.11, *p* = 0.036, $\eta_{p}^{2}$ = 0.21). Indeed, post hoc comparisons confirmed that there was a significant N400 amplitude difference between the two cultural conditions when Chinese participants read statements in Chinese (*t*_19_ = −2.37, *p* = 0.028), but no such difference was found when they read the same statements in English (*t*_19_ = −0.65, *p* = 0.52; Figure 5C). N400 amplitudes also significantly increased for sentences conforming to English culture when participants read them in Chinese as compared with English (*t*_19_ = −3.31, *p* = 0.004; Figure 5C). Topographic maps of the key contrasts showed a widespread negativity mostly affecting central regions of the scalp in all cases (N400) except for Chinese participants reading cultural statements in English. Bayes factor analyses showed that the null hypothesis (i.e., no difference across cultural norms) was around four times more probable than the alternative, when Chinese–English bilinguals read sentences in English (BF_01_ = 3.56), while data favored the alternative hypothesis (i.e., differences across cultural norms) when Chinese–English bilinguals read the same sentences in Chinese (BF_10_ = 2.18).

In order to confirm the robustness of the ERP effects reported above, all analyses were also conducted using the algebraic mean of the mastoid electrodes as reference and, separately, the REST reference.^36^ Despite modest changes in topography, the results were qualitatively unchanged. Also, we investigated ERP mean amplitude in the 550–700 ms window over a group of six centroparietal electrodes (CP1, CPz, CP2, P1, Pz, P2) to evaluate possible contamination of the N400 by the P600 underlying latent component typically observed in overt judgment tasks.^37^, ^38^, ^39^ The result indicated that N400 modulation in the various comparisons made was not differently affected by ERP amplitudes in the P600 window, thus minimizing the risk of spurious effects resulting from P600 contamination (see Figure S1).

## DISCUSSION

We investigated semantic processing in native speakers of English and native speakers of Mandarin Chinese presented with violations of generic knowledge and cultural norms. We expected language of operation to affect violations of cultural norms^3^, ^18^, ^37^ but not violations of generic knowledge. Critically, we sought to determine whether language‐induced cultural shifts in bilinguals coincide with a change in sentence acceptability judgments and N400 modulation across languages, which would indicate that language of operation modulates access to deep‐seated conceptual representations, rather than merely regulating overt, strategic use of cultural knowledge.

First, we found that native speakers of Chinese and native speakers of English process generic knowledge violations in comparable ways, both behaviorally and at a neurophysiological level, independent of language of operation (*Predictions 1* and *2*). This observation reduces de facto the likelihood that the Chinese participants tested here had a shallower understanding of the materials than their British counterparts or vice versa, since N400 modulations have been shown to index the difficulty of retrieval from long‐term semantic memory^19^, ^21^, ^40^ as well as anticipatory top‐down conceptual information processing.^41^, ^42^, ^43^ Recall that we used an overt acceptability judgment task focused on world knowledge evaluation rather than culture in an attempt to equalize task requirements between conditions and control P600 modulations arising from spontaneous stimulus re‐evaluation. Notably, participants were slower in responding to acceptable than unacceptable sentences. This pattern likely reflects the nature of world knowledge processing, where violations are quickly detected, but acceptable sentences require deeper semantic engagement and more thorough evaluation to ensure they do not contain subtle inconsistencies with world knowledge (see, e.g., Pylkkänen et al.^44^). As expected, we observed amplitude shifts by acceptability in the P600 time‐window, but P600 modulation did not interact with group or language in any of the comparisons made, minimizing the risk of N400 contamination by P600 shifts.^45^

Second, we found that violations of cultural norms elicited the predicted reversal in acceptability ratings and N400 modulations between native speakers of Mandarin Chinese and native speakers of English, thus validating symmetrically opposed expectations across groups regarding cultural norms (*Prediction 3*). Consistent with previously reported effects of strong social norm violations,^12^, ^46^, ^47^ the corresponding N400 modulations had a frontocentral distribution in the Chinese participants and a slightly more posterior distribution in the British participants. One original feature of the current study is that the exact same statements were used within‐ and between‐subjects (albeit translated into the other language), such that sentences acceptable from one cultural viewpoint violated culture norms from the other viewpoint. Thus, differences in topography between cultural conditions could hardly be attributed to differences in the stimulus sets used. We note that processes at work when evaluating cultural norms seem markedly different from those involved when participants are faced with violations of their moral beliefs,^39^ judging from the lack of P600 modulation by cultural expectations (Figure S1).

Third, we found an interaction between language of operation and cultural expectation within the same fluent bilinguals, such that the difference in acceptability between cultural norm conditions was reduced when participants read statements in their second language.^3^, ^17^ More specifically, acceptability of British (foreign) culture norms increased in bilinguals when concepts were presented in the second language while acceptability of their native culture norms decreased. Importantly, this was not the result of a speed–accuracy trade‐off because reaction times showed a convergent pattern of modulation featuring the same interaction (even though they were overall slower in English as should be expected). This shift in acceptability is reminiscent of effects found in the foreign language effect literature,^48^ where using a foreign language has been argued to alter cognitive processes such as decision‐making and moral judgments.^49^, ^50^ While a convergence of mechanisms can be expected between the process under study here and the foreign language effect, our study focused on cultural stereotypes as opposed to emotionally challenging decision‐making and thus convergences between fields can only be tentative.

Critically, the cultural shift driven by language was accompanied by a cancellation of N400 modulation when Chinese participants operated in English (*Prediction 4*). The N400 is taken to be an index of semantic–conceptual processing and can be observed in conditions where the input is extremely impoverished^51^, ^52^ or when attentional resources are insufficient for information to be processed consciously.^53^ In other words, we can conclude that the mechanism underlying the shift in perspective prompted by language originated at the level of semantic retrieval rather than at a conscious, re‐evaluation stage of information processing (usually indexed by the P600). Furthermore, the suppression of N400 differences between culture norm conditions in English is unlikely to result from low proficiency in L2, that is a situation whereby the effect would disappear when bilinguals are tested in English simply because they were exposed to translations of the materials in the second language. This is highly unlikely because such an explanation should equally apply to the case of generic knowledge testing. Since the pattern of semantic processing was very similar in Chinese and English in bilingual participants for generic knowledge testing, we can be confident that the difference observed in the case of cultural norm testing is valid. Put simply, our findings can be interpreted as language triggering a conceptual shift rather than merely influencing surface behavior.

We note that behavioral and neurophysiological indices of brain function could have even aligned further, since bilinguals operating in English did not show a complete cancellation of cultural expectations in Chinese as measured by acceptability judgments. Such dissociation is not new,^54^, ^55^, ^56^ but ERP modulation patterns observed in the P600 range suggest that any difference in semantic processing induced by language were soon recovered at the stage of information re‐evaluation since there was no interaction between language of operation and culture norm condition in the P600 range (Supporting Information).

It is worth noting that in the current study the critical words appeared in sentence–final position, which could have contributed to spurious positivity beyond the N400 range. Stowe et al.^57^ have argued that such positivity operates independently from semantic effects, emerging as an additive component that reflects the natural culmination of sentence processing, such as prosodic information integration, perceptual boundary marking, or resolution of ambiguities. However, none of these mechanisms are thought to index spreading of activation in the semantic system and none were directly manipulated in our experimental design. The sentence–final positivity should not have interfered with our ability to detect the N400 effects of interest or compromise the observed pattern of P600 modulations, especially given the lack of interaction in the P600 range.

Our study has several limitations that future research should address. We detected no significant block order effects (English‐first vs. Chinese‐first), but we must keep in mind that such comparison is strongly underpowered given that it involves a between‐subject factor (block order) and a sample size incompatible with the testing of two‐way interactions (e.g., acceptability by block order). Future studies with larger participant samples could look at potential interactions between language block order and semantic processing effects. The generalizability of our findings is also limited by the homogeneity of our participant sample. Participants had similar language dominance profiles, ethnolinguistic backgrounds, and language exposure. While controlling for confounding variables, this fairly homogenous sample restricts our ability to extend conclusions to the broader bilingual population.

Future research should prioritize larger sample sizes to better disentangle order effects from priming effects. The use of a similar paradigm introducing cultural norm violation within sentences rather than in sentence–final position would offer an excellent opportunity for replication of the current results. Additionally, researchers should systematically compare language‐immersed versus nonimmersed contexts to understand how environmental factors shape language processing. Expanding to more diverse bilingual populations (e.g., varying in proficiency levels, age of acquisition, and language use patterns) would provide a more comprehensive understanding of contextual and linguistic influences on language‐modulated culture processing. Finally, investigating additional language pairs beyond Chinese–English would further illuminate how specific linguistic features and cultural associations interact with neurocognitive mechanisms during culture processing.

## CONCLUSION

Our research provides the first evidence for an effect of language of operation on cultural norm perception in bilinguals, manifesting through modulations of acceptability ratings, reaction times, and N400 amplitudes. Such convergence between explicit behavioral measures and neurophysiological indices of semantic processing provides convincing evidence that functioning in a second language disposes one to be more tolerant toward foreign culture values. International relations between nations systematically involve discussions between parties of experts whose foreign language knowledge is often elementary and sometimes inexistent.^58^, ^59^ Our findings may have repercussions for negotiations between nations of markedly different cultures. According to our findings, to experience a genuine cultural understanding at a deep, conceptual level requires one to learn the language of the other culture, not just rely on one's acquaintance with foreign cultural norms. As former UK Secretary of State for Foreign and Commonwealth Affairs William Hague put it: “Diplomacy is the art of understanding different cultures, and using this understanding to predict and influence behavior. Speaking the local language is the essential first step in this process.”^58^

## AUTHOR CONTRIBUTIONS

Jiehui Hu: Conceptualization, investigation, data analysis, visualization, and paper writing and editing; Aina Casaponsa: Data analysis, visualization, and paper writing and editing; Wanyu Zhang: Data analysis, visualization, and paper editing; Rafał Jończyk: Data analysis, visualization, and paper editing; Yan Jing Wu: Conceptualization and paper editing; Shan Gao: Conceptualization and paper editing; Guillaume Thierry: Conceptualization, data analysis, visualization, and paper writing and editing.

## CONFLICT OF INTEREST STATEMENT

The authors declare no conflicts of interest.

## PEER REVIEW

The peer review history for this article is available at https://publons.com/publon/10.1111/nyas.15407.

## Supporting information



Supporting Information

## Data Availability

The data and code used for data analysis in this study are available at Open Science Framework (OSF), 10.17605/OSF.IO/JDG45.
