# Supplementary material for: Foreign cultural norms are better accepted in the second language
Source: Ann N Y Acad Sci. 2025 Aug 4;1551(1):257–69. doi: 10.1111/nyas.15407 (PMC12448271; doi:10.1111/nyas.15407)
Supplement: Supplementary file 1 — Supporting Information [file NYAS-1551-257-s001.docx]

**Supporting Information**

## **Behavioral results**

## **Prediction 1: Comparable generic semantic processing between groups (Fig 2)**

*Maximal models for rates and reaction times as dependent variables (DV) in R syntax:*

*DV ~ acceptability*group + block + (1+acceptability|participant) + (1+group|item)*

**Acceptability.** There was a main effect of acceptability (*χ^2^*_1_ = 123.99, *p* < .001) in the absence of a group effect (*χ^2^*_1_ = .26, *p* = .60**)** or an interaction between acceptability and group (*χ^2^*_1_ = .22, *p*= .64). Planned comparisons confirmed similar rates across groups for acceptable (*β* = .21, *SE* = .40, *z* = .52, *p* = .60) and unexpected (*β* = -.13, *SE* = .58, *z* = -.22, *p* = .83) generic knowledge statements. Further analyses using Bayes Factors (BF_01_) were carried out to compare generic knowledge processing between groups. Results showed that the null hypothesis (i.e., no differences between groups) was around 3 times more probable than the alternative for both type of endings (acceptable: *BF*_01_ = 3.05; unexpected: *BF*_01_ = 3.24).

**Reaction times.** Response times mirrored the pattern of results found for acceptability rates. There was a significant main effect of acceptability (*F*_1, 90_ = 5.05, *p* = .03), showing slower responses for acceptable endings than for endings violating generic semantic knowledge (acceptable: *β* = 1356, *SE* = 70; unexpected: *β* = 1194, *SE* = 59), whilst the effect of group (*F*_1, 45.6_ = .52, *p* = .47), or the interaction between group and acceptability (*F*_1, 61.8_ = .68, *p* = .41) were not significant. Planned comparisons showed no significant differences across groups for either type of sentence completions (acceptable: *β* = 24, *SE* = 127, *t*_47.5_ = .19, *p* = .85; unexpected: *β* = 120, *SE* = 102, *t*_52.1_ = 1.18, *p* = .24). Bayes Factor analyses showed that the null hypothesis (i.e., no differences between groups) was around 3 times more likely than the alternative for acceptable statements (*BF*_01_ = 3.17) and around 2 times more likely for sentences violating generic semantic knowledge (*BF*_01_ = 1.86).

## **Prediction 2: Comparable generic semantic processing between languages in bilinguals (Fig. 3 a, b)**

*Parsimonious models for rates and reaction times in R syntax:*

*DV ~ acceptability*language + block + (1+acceptability:language|participant) + (1+language|item)*

**Acceptability.** Rates were high for acceptable endings (88 %; *β* = 3.11, *SE* = .41) and low for endings violating generic knowledge (6 %; *β* = -4.54, *SE* = .51), resulting in a main effect of acceptability (*χ^2^* = .98.96, p < .001) in the absence of a significant effect of language (*χ^2^* = .86, p = .35), or an interaction between acceptability and language of operation (*χ^2^* = .10, p = .75). Planned comparisons confirmed that bilinguals’ rates were unaffected by language whether or not statements were acceptable (acceptable: *β* = .39, *SE* = .42, *z* = .93, *p* = .35; unexpected: *β* = .12, *SE* = .60, *z* = .21, *p* = .84). In addition, Bayesian Factor analyses revealed that the null hypothesis (i.e., no difference across languages of operation) was around 3 times more probable than the alternative for endings violating generic knowledge (*BF*_01_ = 3.20), however raw data was not sensitive enough to provide evidence against or in favor of the null hypothesis for acceptable endings (*BF*_01_ = .68).

**Reaction times.** Participants were faster in Chinese (*β* = 1239, *SE* = 84) than in English context (*β* = 1697, *SE* = 95) as shown by a main effect of language of operation (*F*_1, 21.9_= 15.81, *p* < .001). There was also a marginal effect of acceptability (*F*_1, 45.1_= 3.83, *p* = .057), such that response to acceptable endings were slower than responses to unexpected endings (acceptable: *β* = 1567, *SE* = 95; unexpected: *β* = 1372, *SE* = 73). The interaction was not significant (*F*_1, 42.3_= .21, *p* = .65). Planned comparisons showed slower reaction times when bilinguals operated in English than in Chinese irrespective of acceptability (acceptable: *β* = 440, *SE* = 125, *t*_24.3_ = 3.53, *p* = .002; unexpected: *β* = 478, *SE* = 120, *t*_24.5_ = 3.97, *p* < .001). Bayesian Factor analyses confirmed that the data was more likely to occur under the alternative hypothesis (i.e., differences across language of operation) for both acceptable (*BF*_10_ = 25.23) and unexpected generic knowledge statements (*BF*_10_ = 59.64).

**Prediction 3: Opposite cultural expectations between groups (Fig. 4)**

*Maximal models for rates and reaction times as dependent variables (DV) in R syntax:*

*DV ~ acceptability*group + block + (1+acceptability|participant) + (1+group|item)*

**Acceptability.** Rates were higher for statements representative of Chinese culture (79%, *β* = 2.16, *SE* = .30) than for statements representative of British culture (73%, *β* = 1.53, *SE* = .26). This resulted in a main effect of cultural background (*χ^2^*_1_ = .6.75 *p* = .009). A main effect of group was also found (*χ^2^*_1_ = 20.91 *p* < .001), as well as and a significant interaction between group and cultural background (*χ^2^*_1_ = 58.96, *p* < .001). Planned comparisons showed that English speakers rated statements representative of the British culture as more acceptable than statements representative of the Chinese culture (*β* = 1.14, *SE* = .44, *z* = 2.60, *p* = .009; British culture: 83%, *β* = 2.37, *SE* = .35; Chinese culture: 69%, *β* = 1.23, *SE* = .36), whilst Chinese speakers rated statements representative of the Chinese culture as more acceptable than statements representative of the British culture (*β* = 2.40, *SE* = .41, *z* = 5.85, *p* < .001; Chinese culture: 89%, *β* = 3.08, *SE* = .38; British culture: 61%, *β* = .69, *SE* = .30). Furthermore, both groups differ in their rates of British culture and Chinese culture statements. That is English speakers rated British culture statements as more acceptable than Chinese bilingual speakers (*β* = 1.68, *SE* = .37, *z* = 4.57, *p* < .001) and Chinese bilingual participants rated Chinese culture statements as more acceptable than English speakers (*β* = 1.85, *SE* = .45, *z* = 4.15, *p* < .001). Bayesian Factor analyses confirmed that the data was more likely to occur under the alternative hypothesis (i.e., differences across groups) for both British culture (*BF*_10_ = 9954.38) and Chinese culture statements (*BF*_10_ = 22879.55). Bayes Factor analyses for the magnitude of the bias towards native cultural norms favored the alternative hypothesis (*BF*_10_ = 8.86e+11).

**Reaction times.** Response latencies analyses showed a significant interaction between group and cultural background (*F*_1, 75.33_ = 25.06, *p* < .001) in the absence of a main effect of group (*F*_1, 45.5_ = 1.82, *p* = .18) or cultural background (*F*_1, 81.85_ = .56, *p* = .46). Planned comparisons revealed that English speakers were faster responding to statements representative of the British culture than to statements representative of the Chinese culture (*β* = 252, *SE* = 82, *t*_81.9_ = 2.48, *p* = .01; British culture: *β* = 1532, *SE* = 124; Chinese culture: *β* = 1785, *SE* = 120), whilst Chinese bilinguals operating in their L1 were faster to respond to statements representative of the Chinese culture than to statements representative of the British culture (*β* = 361, *SE* = 87, *t*_75.6_ = 4.13, *p* < .001; Chinese culture: *β* = 1274, *SE* = 114; British culture: *β* = 1634, *SE* = 118). Furthermore, English and Chinese-English bilingual speakers respond to statements representative of the British culture with similar response latencies (*β* = -102, *SE* = 167, *t*_50.7_ = -.61, *p* = .54; English group: *β* = 1532, *SE* = 124; Chinese-English group: *β* = 1634, *SE* = 118), whilst Chinese-English bilingual speakers responded faster to Chinese culture statements than English speakers (*β* = -511, *SE* = 161, *t*_52.2_ = 3.18, *p* = .003; English group: *β* = 1785, *SE* = 120; Chinese-English group: *β* = 1274, *SE* = 114. Bayesian Factor analyses for the differences across groups favored the null hypothesis for British culture statements (*BF*_01_ = 2.59) and the alternative hypothesis (i.e., differences across groups) for Chinese culture statements (*BF*_10_ = 26.76). Bayes Factor analyses for the magnitude of the bias towards native cultural norms also favored the alternative hypothesis for response latencies (*BF*_10_ = 1.53e+06).

**Prediction 4: Embracing foreign culture in the foreign language (Fig. 5)**

*Parsimonious models for rates and reaction times in R syntax:*

*DV ~ acceptability*language + block + (1+acceptability:language|participant) + (1+language|item)*

**Acceptability.** Acceptability rates for Chinese culture statements (86%, *β* = 2.83, *SE* = .42) were overall higher than for English culture statements (64%, *β* = .89, *SE* = .31; *χ^2^*_1_ = 8.42, *p* = .004). Indeed, English-culture statements were rated as less acceptable than Chinese-culture statements in both English (*β* = -1.24, *SE* = .42, *z* = -2.90, *p* = .004) and Chinese contexts (*β* = -2.64, *SE* = .56, *z* = 4.76, *p* < .001). Analyses also revealed a main effect of language of operation (*χ^2^*_1_ = 5.42, *p* = .02) and an interaction between culture-specific statements and language (*χ^2^*_1_ = 8.88, *p* = .003).

**Reaction times.** Response latencies analyses revealed a main effect of culture-specific knowledge (*F*_1, 76.29_ = 12.83, *p* < .001) and language of operation (*F*_1, 20.19_ = 9.88, *p* = .005) in the absence of a significant interaction (*F*_1, 31.39_ = 2.64, *p* = .11). As expected, participants’ responses were faster in Chinese (*β* = 1456, *SE* = 118) than in English (*β* = 1950, *SE* =128) and for Chinese-culture statements (*β* = 1560, *SE* = 97) than for English-culture statements (*β* = 1845, *SE* = 108).

## **Supporting Table S1** Average acceptability ratings (and standard deviation) obtained in China and in the UK from different groups of participants in 4 rounds of stimulus norming and selection.

|  |  | Generic knowledge | | Cultural norm | |
| --- | --- | --- | --- | --- | --- |
| Norming test |  | Acceptable | Unexpected | Chinese culture | British culture |
|  | Chinese subjects | 1.07 (0.56) | -1.43 (0.66) | 1.03 (0.66) | 0.40 (0.80) |
| Round 1 | English subjects | 1.31 (0.79) | -1.73 (0.43) | 0.23 (1.22) | 1.19 (0.84) |
|  | Chinese subjects | 1.11 (0.50) | -0.92 (0.55) | 1.05 (0.55) | 0.60 (0.7) |
| Round 2 | English subjects | 1.42 (0.64) | -1.72 (0.46) | 0.24 (1.20) | 1.09 (0.86) |
|  | Chinese subjects | 1.12 (0.54) | -1.32 (0.50) | 0.86 (0.64) | -0.22 (0.77) |
| Round 3 | English subjects | 1.40 (0.80) | -1.75 (0.47) | -0.19 (1.05) | 1.29 (0.79) |
|  | Chinese subjects | 1.16 (0.42) | -1.00 (0.54) | 1.13 (0.45) | 0.20 (0.74) |
| Round 4 | English subjects | 1.51 (0.48) | -1.72 (0.48) | -0.12 (1.00) | 1.16 (0.80) |

## **Supporting Table S2**. Means and Standard Derivations (*M ± SD*) of trial number per condition

|  | Unexpected | Acceptable | Chinese culture | British culture |
| --- | --- | --- | --- | --- |
| **Chinese-English bilinguals** |  |  |  |  |
| L1 | 34.7 ± 1.9 | 34.1 ± 1.8 | 34.4 ± 2.1 | 34.6 ± 1.6 |
| L2 | 34.5 ± 1.8 | 33.7 ± 2.3 | 34.1 ± 1.8 | 34.3 ± 1.8 |
| **English natives** |  |  |  |  |
| L1 | 35.3 ± 1.3 | 35.3 ± 1.4 | 35.2 ± 1.4 | 35.3 ± 1.4 |

**Supporting Figure S1**

Event-related potentials elicited by sentence endings in the test of generic knowledge and cultural norms at centroparietal electrodes (CP1, CPz, CP2, P1, Pz, P2) to highlight P600 modulations. (**a**) Bilingual participants tested in Chinese (left) and English (right); (**b**) British participants tested in English.

| 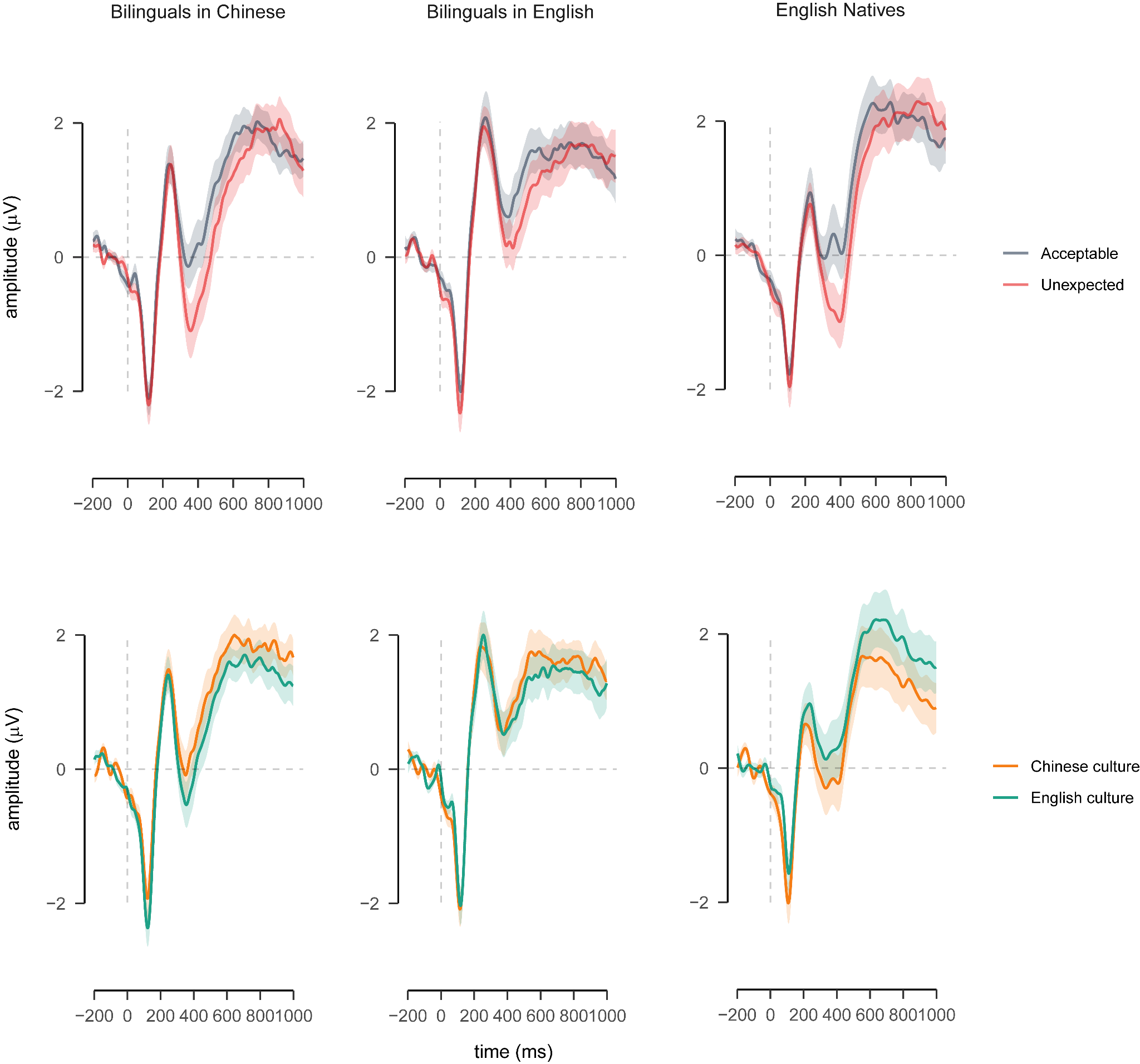 |
| --- |
